# The time is now for action research

**DOI:** 10.1186/2045-4015-2-41

**Published:** 2013-10-23

**Authors:** Sara J Singer

**Affiliations:** 1 Harvard School of Public Health, Kresge Building 3, Room 317, 677 Huntington Avenue, Boston, MA 02115, USA

## Abstract

Despite highly systematic methods for identifying priority problems and assessing intervention effects, the recent study by Tourgeman-Bashkin and colleagues would not be considered rigorous by conventional standards of validity, nor would its sample size of three units impress policymakers eager to promote large-scale change through improvement programs. Yet, study findings suggest that no single intervention would have accomplished as much as the action research approach the authors’ employed. This perspective argues that although action research may lend itself to neither clean comparisons of intervention and control units over time nor far-reaching improvement campaigns, its advantages, including responsiveness to context, emphasis on implementation and sustainability, and insight about underlying mechanisms of change, make rigorous action research a highly attractive alternative for engendering real world improvement.

## 

In Tourgeman-Bashkin and colleagues’ recent article [1], the authors describe a successful action research initiative in which experts in studying the impact of human factors on work processes engaged frontline workers in radiology to identify opportunities for improving patient safety and then planned, implemented, and evaluated interventions to achieve them. Investigators used proactive action research methods to design safety interventions suited to the unique and pressing needs of each unit. Interventions were specific, practical, and often mundane, such as checking each morning for missing information about patients expected that day and calling referring physicians to close the loop in order to reduce the likelihood of wrong person or wrong procedure events. Their research was highly systematic, with extensive observation to identify priority problems and thorough evaluation to assess intervention effects.

This study, however, would not be considered rigorous by conventional standards of validity, nor would its sample size of three units impress policymakers eager to promote large-scale change through improvement programs (see Table 1). Yet, the authors’ results, which revealed different weaknesses in each of the three radiology units in their study, suggest that a one-size-fits-all intervention—no matter how rigorously administered and evaluated—would not have achieved as substantial reductions in potential adverse events as this action research design. Likewise, despite a small sample, lessons drawn from their research are broadly applicable. These contradictions raise fundamental questions about the way we think about quality of health services research design.

**Table 1:** Comparison of strengths and weaknesses of action research compared to randomized controlled trials and improvement campaigns

	**Randomized controlled trial**	**Action research**	**Improvement campaign**
Strengths	• Demonstrates efficacy of an intervention	• Facilitates tailored approach based on context	• Motivates widespread participation
	• Promotes careful attention to intervention protocol	• Allows adjustment	
		• Provides feedback regarding how and why interventions succeed or fail	
		• Engages frontline expertise and increases likelihood of sustaining the intervention	
Weaknesses	• Does not address contextual factors, except through exclusion criteria	• Difficult to assess systematically and to conduct at scale	• Does not address contextual factors

If we value real-world impact, e.g., improvement in patient safety and development of Patient-centered Medical Homes and Accountable Care Organizations, and acknowledge that impact requires effectiveness and not just efficacy, then it may be time to embrace the virtues of action research. A clearer understanding of the advantages of action research may shed light on when it can be most helpful.

### Action research offers three main advantages

#### Responsiveness to context

A key reason that a majority of interventions fail when applied in real world settings is that they do not account for contextual factors, such as resource constraints and fit of the intervention with existing culture and concerns. Action research engages context directly through cooperation between investigators and frontline workers. While this results in inconsistencies in the implementation of an intervention, it ensures that the intervention is responsive to problems on the frontlines.

#### Implementation and sustainability

An additional benefit of engaging frontline workers is the impact of their participation on their willingness to implement and sustain an intervention. While in medicine we eschew the Hawthorne effect as a threat to the validity of experimental research, for managers the discovery of a Hawthorne effect was profoundly positive. It suggested that managers could motivate employees by paying attention to them [2]. These studies spawned a revolutionary shift in management practice [3]. For purposes of solving pressing problems in health care, the Hawthorne effect implies that employees can be motivated to participate in action research and that their involvement will enhance buy-in, which facilitates implementation and promotes sustainability. Rather than being a problem, heightened attention can be an advantage.

#### Insight about mechanisms

A common concern about action research is its scalability. Yet, action research can increase the likelihood of implementing interventions at scale, when the interventions involve complex social and organizational processes. To spur widespread progress toward improvement and organizational transformation, research must convey information about what makes an intervention work and how it varies across units. Through their engagement, action researchers develop a deep understanding about mechanisms (i.e., why a particular intervention is appropriate in a given context and how it is achieving its effect), a grasp of which may facilitate spread. Being able to communicate a logic model, in addition to results, enables more straightforward and thoughtful adoption by others.

In sum, too often, we ask healthcare organizations to adopt too many (albeit evidence-based) interventions, leaving staff overwhelmed and unable to address adequately their most pressing problems. Though action research may lend itself to neither clean comparisons of intervention and control units over time nor to far-reaching improvement campaigns, its advantages make rigorous action research a highly attractive alternative for engendering real world improvement.

### Competing interests

The author declares that she has no competing interests.

### Author information

Sara J. Singer, M.B.A., Ph.D., is an Associate Professor of Health Care Management and Policy at the Harvard School of Public Health and a faculty member in the Department of Medicine at Harvard Medical School in the Mongan Institute for Health Policy, Massachusetts General Hospital. Her research in the field of health care management and policy focuses on how organizational leadership and culture impact efforts to implement health delivery innovations, integrate patient care, and improve performance of health care organizations. Dr. Singer acts as Co-Chair of the Harvard PhD in Health Policy Program Management Track, Implementation Research Director for the Safe Surgery 2015 initiative, and Evaluation Co-Chair for Massachusetts’ Proactive Reduction in Outpatient Malpractice: Improving Safety Efficiency and Satisfaction (PROMISES) program.

### Commentary on

Tourgeman-Bashkin, Osnat, Shinar, David, Donchin, Yoel, Zmora, Ehud, Velleman, Nitsa, Libson, Eugeine, “Radiology department, human factors and organizational perspectives: using action research to improve patient safety”. Israel Journal of Health Policy Research 2013, 2:40.
